# Recognizing nucleosides with transverse electronic transport via perpendicular direction of base planes for DNA sequencing

**DOI:** 10.1186/1556-276X-7-512

**Published:** 2012-09-19

**Authors:** Bing Yang, Ruixin Dong, Xunling Yan, Qiang Shi

**Affiliations:** 1 School of Physics Science and Information Technology, Liaocheng University, Liaocheng Shandong, 252059, China

**Keywords:** DNA, nucleoside, electronic transport, DNA sequencing., 87.15.ag, 87.15.pc, 87.85.Qr

## Abstract

Putting the four DNA nucleosides in the middle of gold [111] nanoelectrodes with base planes parallel to the electrode surface layer, we study the transverse electronic transport properties of four nucleosides along the direction of electrodes. First, the optimal distance of the electrodes is released. The results show that the optimal electrode distance to study transverse electronic transport characteristics of DNA nucleosides is about 0.68 nm. Second, we theoretically calculate the conductance and current of the four nucleosides via perpendicular direction of base planes in the bias range of [−2, 2] V by exploiting the first principle theory. According to the calculated results, we propose three methods to recognize the nucleoside type in practice application.

## Background

A rapid and cost-effective DNA sequencing technique would trigger the revolution of genome-based medical practice and would lead to a new phase of pharmaceuticals [1,2]. It is a promising method to use transverse electronic transport properties across a single-stranded DNA molecule to detect the four nucleobases: adenine (A), cytosine (C), guanine (G), and thymine (T) [3,4]. Since the electronic transport is sensitive to the molecule existing in the interelectrode gap, nucleotide recognition is possible and DNA sequencing is realizable in this way [5-7]. Many researchers have studied on this topic in experimental technology and theoretical calculation, and have pushed forward the technique to practical application [3,8-14]. Most of the studies focus on the transverse conductance of nucleobases or nucleotides in different conditions. Very few studies have looked into transverse charge transport properties of the four nucleosides in DNA along the perpendicular direction of base planes. The four nucleosides, deoxyadenosine (A_0_), deoxycytidine (C_0_), deoxyguanosine (G_0_), and deoxythymidine (T_0_), are the basic units constructing DNA molecules. A study on the transport properties of these nucleosides is helpful for us to understand the DNA's electronic transport properties and to achieve rapid DNA sequencing by physical electronic methods.

In this paper, by exploiting the first principle theory, we construct a computational model theoretically, and calculate and analyze the electronic transport properties of four DNA nucleosides placed in the gold nanoelectrodes along directions perpendicular to the base planes. Based on the calculated results, we propose three methods for DNA sequencing using measured conductance (*G*) and current (*I*).

## Methods

**Figure 1:**
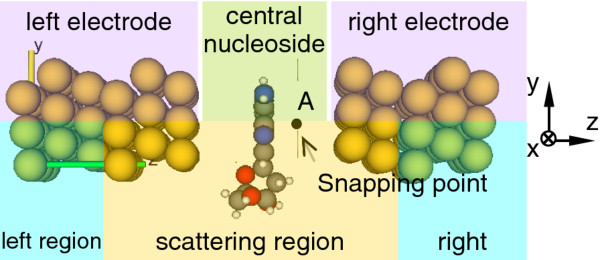
Configuration of the calculation model.

**Figure 2:**
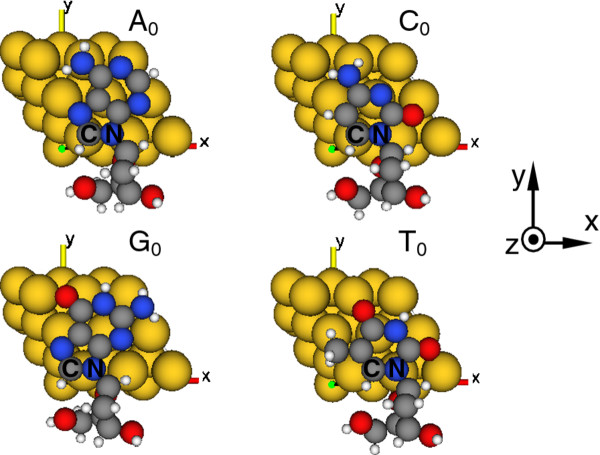
Positions of nucleosides between electrodes.

Our computational models are schematically shown in Figures 1 and 2. In Figure 1, the left and right electrodes are two semi-infinite gold leads with 3 × 3 = 9 atoms, with each layer arranged in the [111] surface geometry along the *z*-axis. The central nucleoside (A_0_, C_0_, G_0_, or T_0_) is placed in the middle point of the electrode gap with the base plane parallel to electrode surfaces. The Cartesian coordinate is also presented in Figure 1. This configuration of the calculation model can be divided into three parts: the left, right, and scattering regions. Both left and right regions are gold electrode bodies. The scattering region comprises three layers (27 atoms) and two layers (18 atoms) of left and right electrode surfaces, respectively, and also of the central nucleoside, as can be seen in the figure. The portion of the electrodes included in the scattering region is to screen out the perturbation from the scattering part to gold electrode body atoms.

The positions of the four nucleosides between electrodes are schematically shown in Figure 2 as viewed from the snapping point A marked in Figure 1 with direction to the left electrode. Considering that the structure and size of nucleosides are different and the pentose sugar ring in the nucleosides cannot be accommodated in the electrode gap, we adjust the nucleoside position so that the vast majority of the base body can be included in the scale of electrode surface, and expectantly, the configuration would give the maximal value of electronic transport. In the calculations, we coordinate one of the nitrogen atoms in the base (blue-colored ball marked with capital letter N) at (0.3, 0.1)-, (0.3, 0.1)-, (0.2, 0.1)-, and (0.4, 0.1)-nm positions in the *x* and *y* planes for A_0_, C_0_, G_0_, and T_0_ nucleosides, respectively, and keep one of their adjacent carbon atoms (gray-colored ball marked with capital letter C) as the same *y*-coordinate figure as that of the pointed nitrogen atom throughout the calculation process, as shown in Figure 2 schematically; we call this configuration as our ‘calculation model’. Our further calculations show that changes of electronic transport of the nucleosides are small and cannot affect the recognition process when the nucleosides have a translational motion within the scale [−0.1, 0.1] nm along the *x*-axis and [−0.05, 0.05] nm along the *y*-axis in the *x* and *y* planes relative to the position of our calculation model. Thus, in the following calculation, we use the calculation model as our research object.

The optimization of the four nucleosides is performed using the linear combination of atomic orbital basis SIESTA code [15,16], implementing the density functional theory (DFT) with the generalized gradient approximation (GGA) and the function of Perdew, Burke, and Ernzerh known as PBE [17,18]. The finite range of the orbital is defined by the orbital confinement energy of 50 meV. A double zeta basis plus polarization (DZP) orbital basis set is used for all the atoms. The resolution of the real-space mesh is defined by a 150Ry cutoff to assure energy and force convergence. The tolerance in the maximum density matrix difference is 10^−5^, and the tolerance in the maximum atomic force is 0.04 eV/Å.

The electronic transport properties are calculated using the first principle quantum transport calculations, performed by the commercially available Atomistix ToolKit, based on the nonequilibrium Green function (NEGF) approach on top of the DFT, within the GGA [19,20]. Good agreement between the theoretical calculations and the experiment data on electron transport in the molecular system has been obtained previously [21,22]. In the study, DZP basis sets are used for H, C, N, and O atoms, while a DZ basis set is used for the gold atom. Core electrons are represented by norm-conserving pseudopotentials using the Troullier-Martins parameterization [23].

## Results and discussion

### Optimal distance of the electrodes

**Figure 3:**
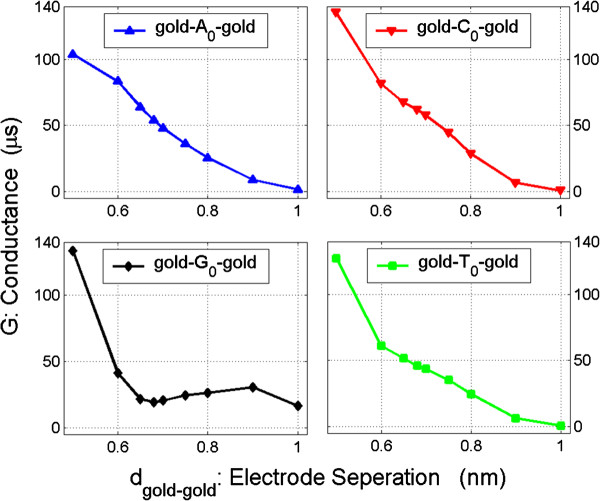
**Conductance (*G*) vs. electrode separation (*d***_**gold-gold**_**) for gold-*X***_**0**_**-gold configurations at zero bias. ***X*_0_ = A_0_, C_0_, G_0_, and T_0_.

For the configurations investigated here, the electronic transport is expected to decrease with the increase of the gold-gold electrode separation. In order to verify this correlation, we calculate the conductance of the configuration with the four nucleosides between the electrodes respectively as separation of electrodes changes when the configuration is biased at zero voltage. The results of conductance (*G*) vs. electrodes separation (*d*_gold-gold_) are shown in Figure 3. Obviously, the transport decays rapidly with electrode separation, increasing in gold-*X*_0_-gold junctions (*X*_0_ = A_0_, C_0_, and T_0_). For the junction gold-G_0_-gold, the conductance remains on almost a platform region from 0.65 to 0.9 nm of electrode separations. This is because of the structure of the G_0_ nucleoside and the interaction between the G_0_ and gold electrodes. Its detailed reason is out of this paper's scope and is not discussed here. In general, the conductance increases with the decrease of electrode separation for all the four configurations. Thus, we can decrease separation of the electrodes to increase transmission of the junctions. However, the separation needs to be large enough to accommodate the four nucleosides (A_0_, C_0_, G_0_, and T_0_). In some hypothetical and fully operational devices, the electrodes are fixed so that we have to find a separation which is optimal for all the four nucleosides. In particular, the electrode gap should be wide enough to accommodate also the non-plane methyl group on T_0_.

**Figure 4:**
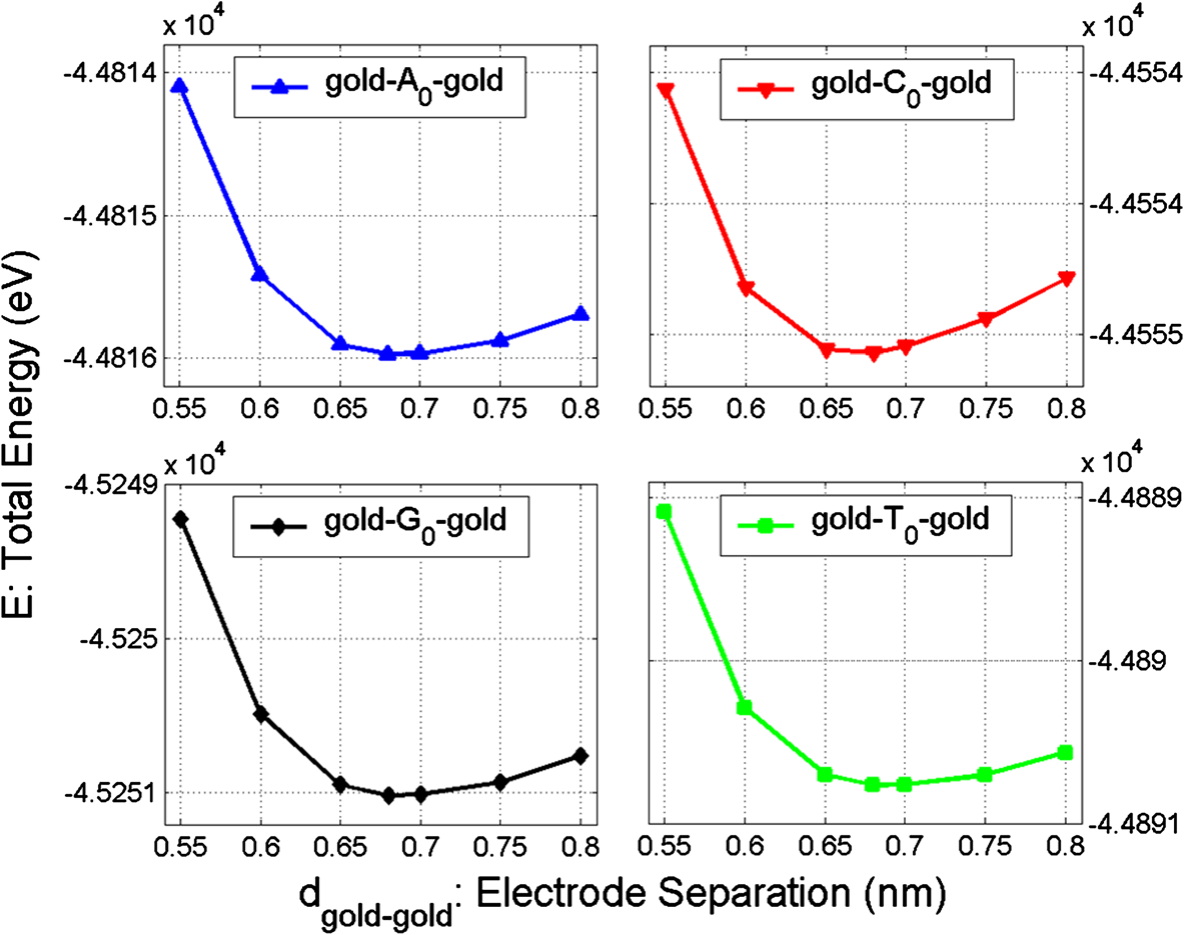
**Total energy (*E*) as function of electrode separation (*d***_**gold-gold**_**) for gold-*X***_**0**_**-gold configurations.***X*_0_ = A_0_, C_0_, G_0_, and T_0_.

In order to determine the optimal separation of the electrodes, we fix the central nucleoside in the lateral position (in the *x* and *y* planes) and change the separation of the electrodes, always keeping the molecule in the middle of the gap, and then we calculate the total energies (*E*) of the junctions for the four nucleosides as functions of electrode separation. The results are shown in Figure 4.

From Figure 4, it is clear that the smallest possible separation of the electrodes is approximately 0.68 nm since there is a notable increase of the total energy for closer separations regardless of the nucleoside type. Considering that we should use a common separation for all four configurations, in the subsequent calculations, we assume that the separation of electrodes is 0.68 nm and that the nucleoside is located in the middle plane of the electrode gap; we denote this configuration as the optimal configuration.

### *G*-*V* and *I*-*V* characteristics and recognizing methods

**Figure 5:**
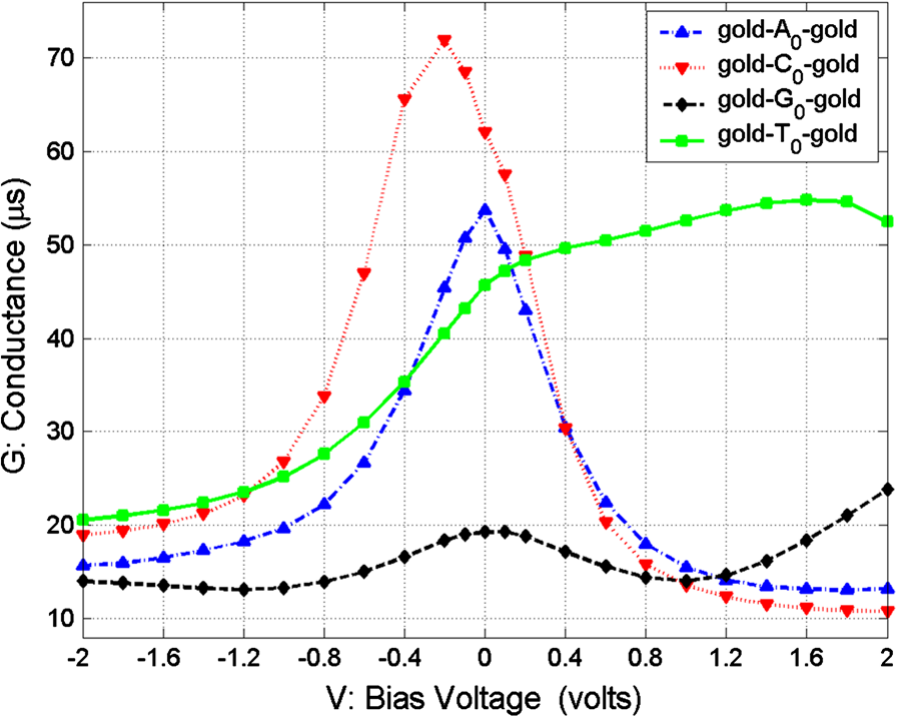
**Conductance (*G*) as function of bias voltage (*V*) from −2 to 2 V.**

**Figure 6:**
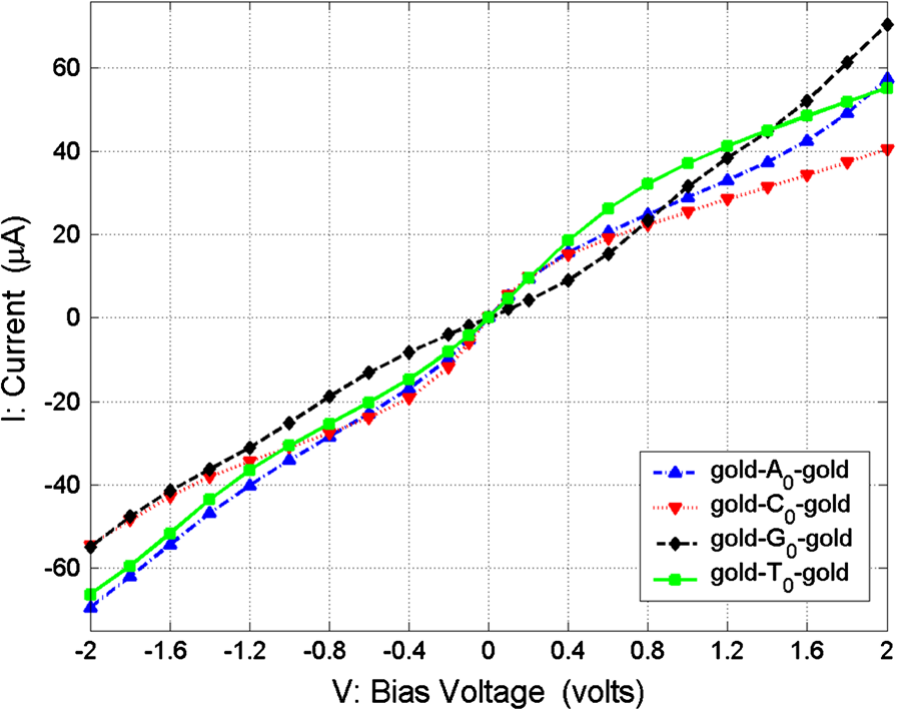
**Current (*I*) as function of bias voltage (*V*) from −2 to 2 V.**

In order to study the transverse electronic transport properties of the four nucleosides, we examine the *G* and *I* properties of the four junctions at the optimal configurations biased in low voltage. Figures 5 and 6 show the *G*-*V* (conductance vs. bias voltage) and *I*-*V* (current vs. bias voltage) curves, respectively, in the moderate range [−2, 2] V.

In order to determine the electrode's contribution to conductance and current, we also calculate the transport of the gold-gold electrode through vacuum with a distance of 0.68 nm biased in the range [−2, 2] V and find that the values are extremely small (*G* ~ 10^−13^ μS and *I* ~ 10^−14^ to 10^−13^ μA). Thus, the electronic transports through the junctions are determined by the nucleosides between the electrodes. Therefore, in the following discussion, we consider that the conductance and current of the nucleoside are the same as those of the junctions respectively, that is, $G\left(gold-X_{0}-gold\right)=G\left(X_{0}\right)$ and $I\left(gold-X_{0}-gold\right)=I\left(X_{0}\right)$, where *X*_0_ can be A_0_, C_0_, G_0_, and T_0_ nucleosides.

From Figures 5 and 6, we can see the conductance and current of the four nucleosides vary with the change of the bias voltage, and their values are not well symmetrical with the zero bias. These asymmetries of the conductance and current are due to the asymmetries of the structure of nucleosides containing the deoxyribose which is not symmetrical in our calculation model.

**Figure 7:**
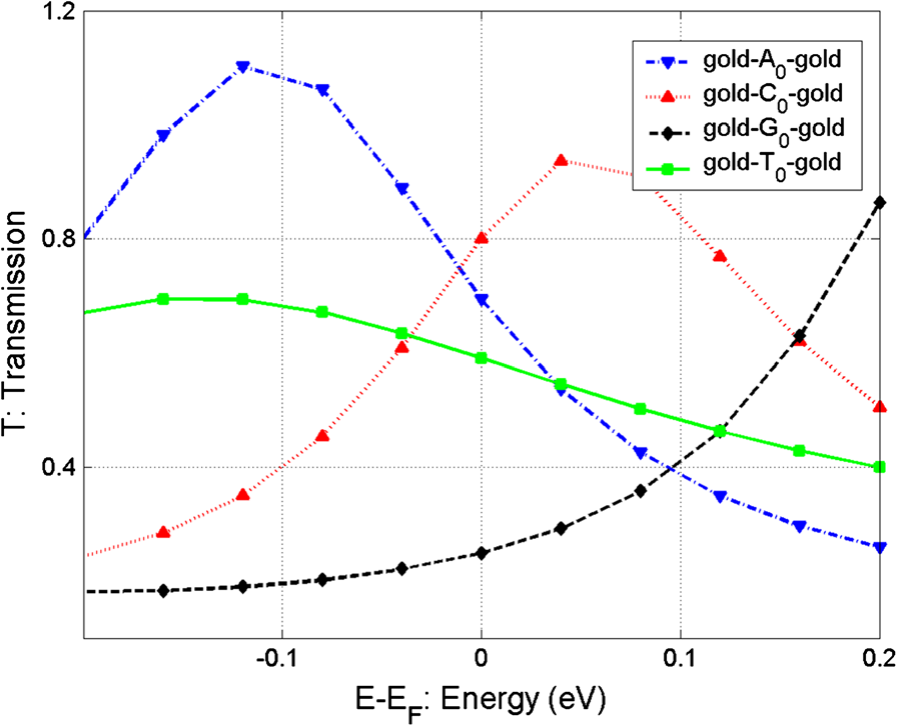
**Transmission coefficient (*T*) spectrum as function of energy (*E*-*E***_**F**_**) for the four junctions.**

In Figure 5, we can see that the conductance of the four nucleosides are well distinguishable when bias is 0 V and when the magnitudes of the conductance are in the order of *G*(C_0_) > *G*(A_0_) > *G*(T_0_) > *G*(G_0_). Figure 7 shows the transmission coefficients as energy function of the four nucleosides in the zero bias condition. Obviously, the transmissions in the *E*_F_ position are in the order of *T*(C_0_) > *T*(A_0_) > *T*(T_0_) > *T*(G_0_), which is the same as the orders of conductance for the four nucleosides. This is because the linear response (zero bias) conductance, *G*, is simply *G* = *G*_0_*T*(*E*_F_), where *G*_0_ = 2*e*^2^/*h* is the quantum conductance, *e* is the electronic charge, and *h* is the Planck constant.

According to the calculated results, we propose several ways to recognize nucleosides between the two gold electrodes. The first method is only to measure the conductance of nucleosides in the zero bias condition. From Figure 5, we can see that conductance of the four nucleosides is separated from each other with the interval of at least 8 μS which can now be easily detected by experiments. Hence, in this method, we can measure the conductance of the nucleoside between the electrodes and determine the nucleoside type according to the relative value relations.

**Figure 8:**
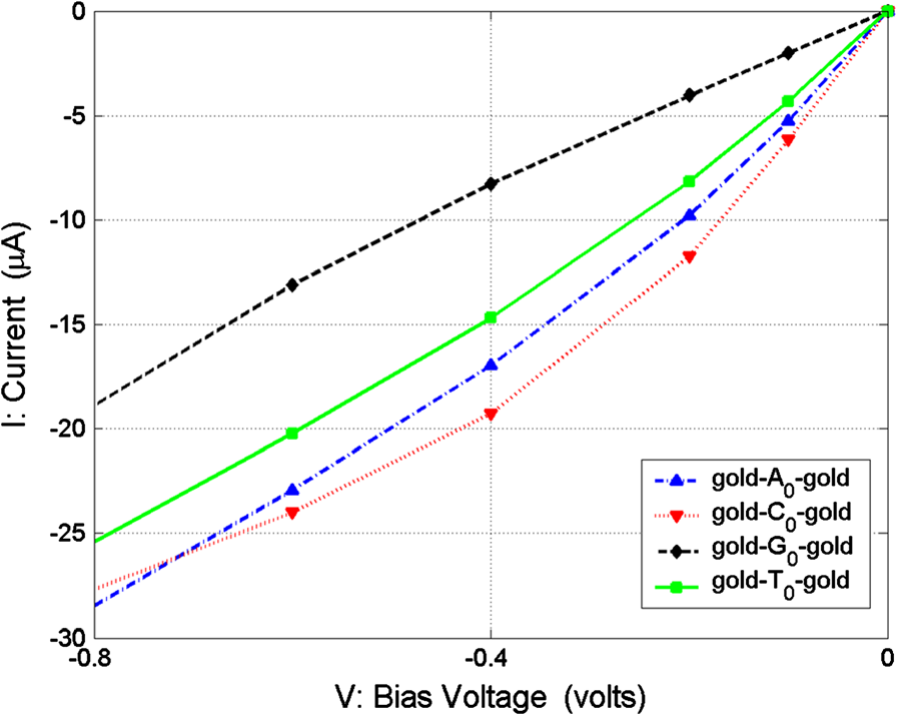
**Current (*I*) as function of bias voltage (*V*) from −0.8 to 0 V for four junctions.**

The second method is only to measure the currents of the nucleosides. From Figure 6, we can see that currents of the four nucleosides are monotonic in their change, with variations in their bias voltage, and the absolute value magnitudes are in the order of *I*(C_0_) > *I*(A_0_) > *I*(T_0_) > *I*(G_0_) in the bias range of [0, −0.6] V. For more clarity, in Figure 8, we show the currents of the four nucleosides in the bias range of [0, −0.8] V. The calculated currents of the four nucleosides are with the interval of microampere order which can now be easily distinguished by experimental apparatus, so in this low bias range, we can measure the currents of nucleosides between the electrodes and recognize the nucleoside type according to the relative current value relations.

**Figure 9:**
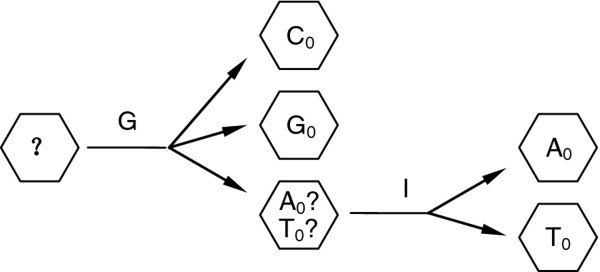
Flow diagram illustration of nucleoside identification in bias range of [0, −0.8] V.

The third method is to measure conductance and current at the same time. From Figure 5, it is clear that the conductance values of C_0_ and G_0_ are the maximum and the minimum, respectively, in the four nucleosides in the negative bias range of [0, −0.8] V and are well separated from the other two nucleosides, A_0_ and T_0_, in this region. From Figure 6, or Figure 8, we can see that the absolute current value of A_0_ is always larger than that of T_0_ for about several microamperes in the bias range of [0, −0.8] V. Hence, in this method, according to the measured conductance, we can distinguish C_0_ and G_0_ from A_0_ and T_0_, and then according to the measured current, we can then distinguish A_0_ and T_0_, as diagrammatically represented in Figure 9.

**Figure 10:**
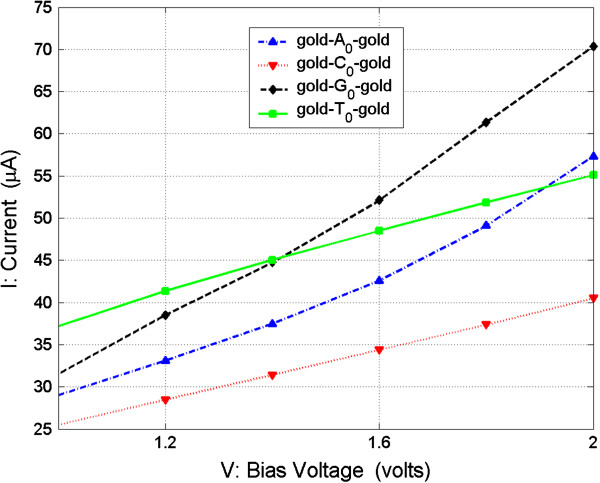
**Current (*I*) as function of bias voltage (*V*) from 1 to 2 V for four junctions.**

Using this method, the nucleosides can be recognized in another bias region of [1,2] V. From Figure 5, it can be seen that the conductance of T_0_ is several times larger than the other three nucleosides, and it is easy to distinguish it from the others. Also, from Figure 6, we can see that the currents of the other three nucleosides C_0_, A_0_, and G_0_ are monotonic in their increase with the increase of bias and that the magnitudes of currents are in the order of *I*(G_0_) > *I*(A_0_)) > *I*(C_0_) in the range of [1,2] V. For more clarity, we show the currents of the four nucleosides in the bias range of [1,2] V in Figure 10.

**Figure 11:**
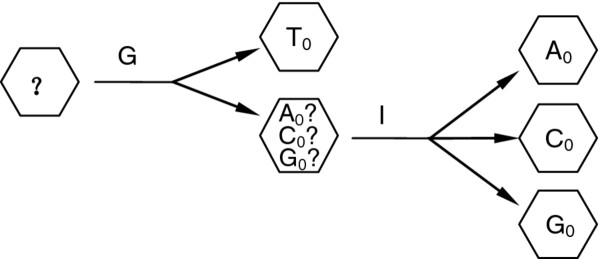
Flow diagram illustration of nucleoside identification in bias range of [1, 2] V.

It is clear that the current interval among the three nucleosides increases from several microamperes to more than a dozen microamperes as the bias voltage increases from 1 to 2 V. Hence, in this method, according to the measured conductance, we can detect the T_0_ from A_0_, C_0_, and G_0_, and according to the measured current, we can then distinguish A_0_, C_0_, and G_0_, as diagrammatically represented in Figure 11.

## Conclusions

In summary, gold electrodes are released in the research experiment in order for the transport property of the four nucleosides of DNA molecule to be studied. Three methods are proposed to recognize them according to the calculated conductance and current. The first method is to measure the conductance of the four nucleosides at zero bias condition. The second method is to measure the current of these nucleosides in a low bias range of [0, −0.6] V. The third is to use the conductance and current measured at the same time. The values of conductance and current in our methods are all with the interval of microsiemens and microampere orders which can now be easily distinguished by experimental apparatus. Therefore, it is possible to recognize the four nucleosides by the protocols proposed in this article.

## Abbreviations

A_0_: deoxyadenosine; C_0_: deoxycytidine; G_0_: deoxyguanosine; T_0_: deoxythymidine.

## Competing interests

The authors declare that they have no competing interests.

## Authors’ contributions

BY calculated the transport properties of the four nucleosides and drafted the manuscript. RD participated in the design of the study and helped draft the manuscript. XY participated in the calculation and helped perform the analysis. QS participated in the figure drafting and helped draft the manuscript. All authors read and approved the final manuscript.

## Authors’ information

BY is an associate professor at the School of Physics Science and Information Technology in Liaocheng University, Shandong, China. He completed his MS degree in the College of Science in Shanghai University, Shanghai. His research interests include the preparation and characterization of DNA-based nanowires using STM and AFM technologies and the simulation and experimental measurement of the electrical conductivity of these nanowires. RD is a professor at the School of Physics Science and Information Technology in Liaocheng University, Shandong, China. She received her doctorate degree in biomedicine from the University of Electronic Science and Technology of China. Her research interests include the conductivity of DNA molecules and the memristor of nanometer devices. XY is a professor at the School of Physics Science and Information Technology in Liaocheng University, Shandong, China. He received his MS degree in Zhengzhou University. His research interests include the conductivity of DNA and the measurement of laser Raman spectrum. QS is a lecturer at the School of Physics Science and Information Technology in Liaocheng University, Shandong, China. He finished his MS degree from Beihang University. His research interests include the magnetism, conductivity, and luminescence properties of thin films and nanomaterials.
